# Dimethyl (*E*)-2-(*N*-phenyl­acetamido)­but-2-enedioate

**DOI:** 10.1107/S1600536810050890

**Published:** 2010-12-11

**Authors:** Shui Liang Guo, Chen Fu, Ting Bin Wen

**Affiliations:** a Department of Chemistry, College of Chemistry and Chemical Engineering, Xiamen University, Xiamen 361005, Fujian, People’s Republic of China

## Abstract

The title compound, C_14_H_15_NO_5_, was obtained from the reaction of acetanilide with dimethyl acetyl­enedicarboxyl­ate in the presence of potassium carbonate. The C=C double bond adopts an *E* configuration and the geometry around the amide N atom is almost planar rather than pyramidal (mean deviation of 0.0032 Å from the C_3_N plane). The packing of the mol­ecules in the crystal structure is stabilized by inter­molecular C—H⋯O hydrogen bonds.

## Related literature

For background to the hydro­amidation of alkynes, see: Severin & Doye (2007) ▶; Goossen *et al.* (2005 ▶); Cacchi & Fabrizi (2005) ▶; For structurally related compounds, see: Kawahara *et al.* (1989 ▶); Penney *et al.* (1995 ▶); Yet *et al.* (2003 ▶); Hua *et al.* (2003 ▶).
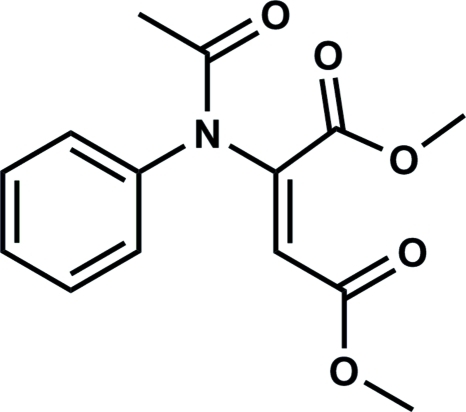


## Experimental

### 

#### Crystal data

C_14_H_15_NO_5_
*M* *_r_* = 277.27
Monoclinic, 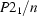

*a* = 9.7920 (5) Å
*b* = 12.1917 (4) Å
*c* = 12.2281 (6) Å
β = 112.629 (6)°
*V* = 1347.42 (11) Å^3^
*Z* = 4
Mo *K*α radiation
μ = 0.11 mm^−1^
*T* = 173 K
0.15 × 0.12 × 0.10 mm

#### Data collection

Oxford Diffraction Gemini S Ultra diffractometer
Absorption correction: multi-scan (*CrysAlis RED*; Oxford Diffraction, 2008 ▶) *T*_min_ = 0.885, *T*_max_ = 1.000
7263 measured reflections
3009 independent reflections
2415 reflections with *I* > 2σ(*I*)
*R*_int_ = 0.029

#### Refinement

*R*[*F* ^2^ > 2σ(*F* ^2^)] = 0.035
*wR*(*F* ^2^) = 0.091
*S* = 1.00
3009 reflections
181 parameters
H-atom parameters constrained
Δρ_max_ = 0.24 e Å^−3^
Δρ_min_ = −0.21 e Å^−3^

### 

Data collection: *CrysAlis CCD* (Oxford Diffraction, 2008 ▶); cell refinement: *CrysAlis RED* (Oxford Diffraction, 2008 ▶); data reduction: *CrysAlis RED*; program(s) used to solve structure: *SHELXTL* (Sheldrick, 2008 ▶); program(s) used to refine structure: *SHELXTL*; molecular graphics: *SHELXTL*; software used to prepare material for publication: *SHELXTL*.

## Supplementary Material

Crystal structure: contains datablocks I, global. DOI: 10.1107/S1600536810050890/zl2334sup1.cif

Structure factors: contains datablocks I. DOI: 10.1107/S1600536810050890/zl2334Isup2.hkl

Additional supplementary materials:  crystallographic information; 3D view; checkCIF report

## Figures and Tables

**Table 1:** Hydrogen-bond geometry (Å, °)

*D*—H⋯*A*	*D*—H	H⋯*A*	*D*⋯*A*	*D*—H⋯*A*
C4—H4*C*⋯O3^i^	0.96	2.53	3.0831 (16)	117
C14—H14*A*⋯O3^ii^	0.93	2.57	3.2016 (15)	125
C12—H12*A*⋯O5^iii^	0.93	2.51	3.3073 (15)	145

Symmetry codes: (i) 
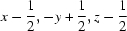
; (ii) 
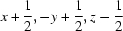
; (iii) 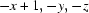.

**Fig. 1.**
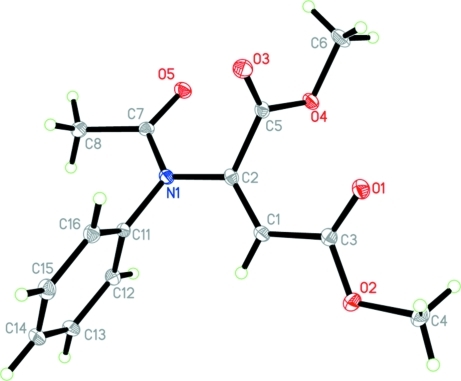
The molecular structure of the title compound with the atom-labelling scheme, showing 30% probability displacement ellipoids.

**Fig. 2.**
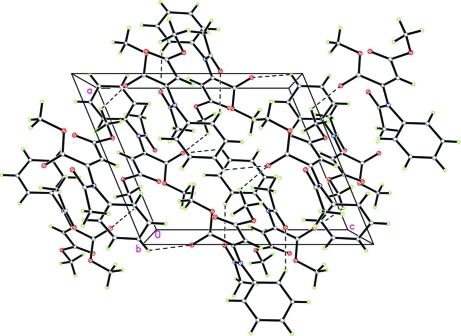
The packing of the molecules, viewed down the b axis. The C—H···O hydrogen bond interactions are shown as dashed lines.

## supplementary crystallographic information

### Comment

Hydroamidation of alkynes has proved to be an effective approach to construct enamides (Severin & Doye, 2007; Goossen *et al.* 2005; Cacchi & Fabrizi 2005), which are important substructures often found in natural products and synthetic drugs (Yet *et al.* 2003). In our studies on the reaction of dimethyl acetylenedicarboxylate with acetanilide in the presence of potassium carbonate, the title compound was formed *via* base mediated hydroamidation.

An X-ray diffraction study has been carried out to determine the structure (Fig. 1). The C=C double bond adopts an E configuration. The geometry around the amide N atom is planar rather than pyramidal, as reflected by the small mean deviation of 0.0032 Å from the least-squares plane defined by the four constituent atoms N1, C2, C7 and C11, which is probably due to the large degree of conjugation between the amide N atom and the adjacent acetyl group (the maximium deviation from the least-squares plane defined by N1, C2, C7, C11 and O5 is 0.0956 (9) Å for N1) (Penney, *et al.* 1995). The C1-C2 double bond is slightly tilted against one ester group with a dihedral angle of only 9.10 (21)° between the (C2, C1, C3) plane and the (C1, C3, O1, O2) plane, but it is tilted against the other ester group with a dihedral angle of 80.25 (4)° between the (C1, C2, C5) plane and the (C2, C5, O3, O4) plane. The dihedral angle of the double bond plane (C1, C2, N1) with respect to the amide group plane (C2, N1, C7, C11) is 23.97 (18) °. The structural features of the title compound agree well with that of similar compounds reported in literature (Kawahara *et al.* 1989; Hua *et al.* 2003).

The packing of molecules in the crystal structure is stabilized by non-classical intermolecular C—H···O hydrogen bonds (Fig. 2, Table 1). The intermolecular hydrogen bonding interactions between O3 atom of the ester group and methyl C-H (C4—H4C···O3^i^) as well as the aromatic C-H (C14—H14A···O3^ii^) form a 2-D networks parallel to the *ac* plane, which is further cross-linked by a hydrogen bond between O5 of the other ester group and an aromatic C-H (C12—H12A···O5^iii^) to give a 3-D hydrogen bonding network (Symmetry codes: (i) x-1/2,-y+1/2,z-1/2; (ii) x+1/2,-y+1/2,z-1/2; (iii) -x+1,-y,-z).

### Experimental

To a solution of acetanilide (0.27 g, 2.00 mmol) and dimethyl acetylenedicarboxylate (0.29 g, 2.04 mmol) in toluene (10 ml), potassium carbonate (0.57 g, 4.13 mmol) was added at room temperature. The mixture was then refluxed for 12 h under an atmosphere of dinitrogen. After concentration, the residue was purified by flash chromatography (ethyl acetate/petroleum = 1:2) to give the product as a white solid. Yield: 0.37 g, 67.2%. ^1^H NMR (400 MHz, CDCl_3_): δ 7.60-7.24 (m, 5 H, Ar), 5 .85 (s, 1 H, CH), 3.79 (s, 3 H, OCH_3_), 3.58 (s, 3 H, OCH_3_), 1.96 (s, 3 H, CH_3_) ppm; ^13^C NMR (101 MHz, CDCl_3_): δ 168.6, 166.4, 166.1, 152.3, 135.9, 129.6, 123.2, 121.5, 107.5, 52.8, 52.0, 20.8 ppm. ESI-MS: 300.3 [*M*+Na]+. Single crystals were obtained by slow evaporation of a solution in dichloromethane/hexane.

### Refinement

One of the reflections, (-5 3 5), was found to be inconsistent with an I(obs) value more that 10 times SigmaW diffeent from I(calc). Inspection of the frame showed that the reflection was located at the frame edge and it was thus omitted from the refinement.

All non-hydrogen atoms were refined anisotropically. The hydrogen atoms were positioned geometrically (C—H = 0.93, 0.93 or 0.96Å for phenyl, methylene or methyl H atoms respectively) and included in the refinement in the riding model approximation. The displacement parameters of vinyl and phenyl H atoms were set to 1.2*U*_eq_(C), while those of methyl H atoms were set to 1.5*U*_eq_(C). In the final Fourier map the highest peak is 0.72 Å from atom H8A and the deepest hole is 0.59 Å from atom C8.

### Crystal data

**Table:**

C_14_H_15_NO_5_	*F*(000) = 584
*M**_r_* = 277.27	*D*_x_ = 1.367 Mg m^−^^3^
Monoclinic, *P*2_1_/*n*	Mo *K*α radiation, λ = 0.71073 Å
Hall symbol: -P 2yn	Cell parameters from 4186 reflections
*a* = 9.7920 (5) Å	θ = 2.8–29.0°
*b* = 12.1917 (4) Å	µ = 0.11 mm^−^^1^
*c* = 12.2281 (6) Å	*T* = 173 K
β = 112.629 (6)°	Block, colorless
*V* = 1347.42 (11) Å^3^	0.15 × 0.12 × 0.10 mm
*Z* = 4	

### Data collection

**Table:**

Oxford Diffraction Gemini S Ultra diffractometer	3009 independent reflections
Radiation source: Enhance (Mo) X-ray Source	2415 reflections with *I* > 2σ(*I*)
graphite	*R*_int_ = 0.029
Detector resolution: 16.1930 pixels mm^-1^	θ_max_ = 27.5°, θ_min_ = 2.8°
ω scans	*h* = −12→12
Absorption correction: multi-scan (*CrysAlis RED*; Oxford Diffraction, 2008)	*k* = −15→10
*T*_min_ = 0.885, *T*_max_ = 1.000	*l* = −14→15
7263 measured reflections	

### Refinement

**Table:**

Refinement on *F*^2^	Primary atom site location: structure-invariant direct methods
Least-squares matrix: full	Secondary atom site location: difference Fourier map
*R*[*F*^2^ > 2σ(*F*^2^)] = 0.035	Hydrogen site location: inferred from neighbouring sites
*wR*(*F*^2^) = 0.091	H-atom parameters constrained
*S* = 1.00	*w* = 1/[σ^2^(*F*_o_^2^) + (0.058*P*)^2^] where *P* = (*F*_o_^2^ + 2*F*_c_^2^)/3
3009 reflections	(Δ/σ)_max_ = 0.001
181 parameters	Δρ_max_ = 0.24 e Å^−^^3^
0 restraints	Δρ_min_ = −0.21 e Å^−^^3^

### Special details

**Table:**

Geometry. All esds (except the esd in the dihedral angle between two l.s. planes) are estimated using the full covariance matrix. The cell esds are taken into account individually in the estimation of esds in distances, angles and torsion angles; correlations between esds in cell parameters are only used when they are defined by crystal symmetry. An approximate (isotropic) treatment of cell esds is used for estimating esds involving l.s. planes.
Refinement. Refinement of F^2^ against ALL reflections. The weighted R-factor wR and goodness of fit S are based on F^2^, conventional R-factors R are based on F, with F set to zero for negative F^2^. The threshold expression of F^2^ > 2sigma(F^2^) is used only for calculating R-factors(gt) etc. and is not relevant to the choice of reflections for refinement. R-factors based on F^2^ are statistically about twice as large as those based on F, and R- factors based on ALL data will be even larger.

### Fractional atomic coordinates and isotropic or equivalent isotropic displacement parameters (Å^2^)

**Table:**

	*x*	*y*	*z*	*U*_iso_*/*U*_eq_	
O1	0.35048 (9)	0.34762 (7)	0.14129 (7)	0.0233 (2)	
N1	0.65965 (10)	0.10602 (8)	0.11896 (8)	0.0173 (2)	
C1	0.50153 (12)	0.26233 (10)	0.05139 (10)	0.0184 (2)	
H1A	0.5426	0.2750	−0.0045	0.022*	
O2	0.36589 (9)	0.41845 (7)	−0.02353 (7)	0.0233 (2)	
C2	0.54018 (12)	0.17090 (9)	0.11658 (10)	0.0166 (2)	
O3	0.53999 (10)	0.14493 (7)	0.30872 (7)	0.0265 (2)	
C3	0.39784 (12)	0.34379 (9)	0.06335 (10)	0.0184 (2)	
O4	0.33137 (9)	0.10799 (7)	0.15035 (7)	0.0211 (2)	
C4	0.27339 (14)	0.50801 (10)	−0.01660 (12)	0.0268 (3)	
H4A	0.2562	0.5569	−0.0821	0.040*	
H4B	0.3218	0.5470	0.0563	0.040*	
H4C	0.1806	0.4797	−0.0194	0.040*	
O5	0.56257 (9)	−0.04796 (7)	0.16290 (8)	0.0233 (2)	
C5	0.47167 (13)	0.13911 (9)	0.20343 (10)	0.0185 (3)	
C6	0.26254 (15)	0.07359 (11)	0.22997 (12)	0.0295 (3)	
H6A	0.1618	0.0529	0.1850	0.044*	
H6B	0.2646	0.1330	0.2821	0.044*	
H6C	0.3154	0.0120	0.2757	0.044*	
C7	0.66568 (12)	−0.00447 (9)	0.14735 (10)	0.0179 (2)	
C8	0.80390 (13)	−0.06526 (11)	0.15872 (11)	0.0251 (3)	
H8A	0.7954	−0.1405	0.1783	0.038*	
H8B	0.8875	−0.0324	0.2201	0.038*	
H8C	0.8171	−0.0617	0.0850	0.038*	
C11	0.77404 (12)	0.15731 (9)	0.08915 (10)	0.0167 (2)	
C12	0.77754 (13)	0.14120 (10)	−0.02186 (10)	0.0195 (3)	
H12A	0.7062	0.0980	−0.0778	0.023*	
C13	0.88875 (13)	0.19028 (10)	−0.04866 (11)	0.0224 (3)	
H13A	0.8931	0.1790	−0.1225	0.027*	
C14	0.99298 (13)	0.25583 (10)	0.03413 (11)	0.0247 (3)	
H14A	1.0673	0.2887	0.0159	0.030*	
C15	0.98695 (13)	0.27261 (10)	0.14413 (11)	0.0240 (3)	
H15A	1.0568	0.3173	0.1993	0.029*	
C16	0.87727 (13)	0.22307 (10)	0.17251 (11)	0.0208 (3)	
H16A	0.8733	0.2339	0.2465	0.025*	

### Atomic displacement parameters (Å^2^)

**Table:**

	*U*^11^	*U*^22^	*U*^33^	*U*^12^	*U*^13^	*U*^23^
O1	0.0234 (5)	0.0234 (5)	0.0264 (5)	0.0029 (4)	0.0133 (4)	−0.0002 (4)
N1	0.0167 (5)	0.0172 (5)	0.0206 (5)	0.0008 (4)	0.0101 (4)	0.0014 (4)
C1	0.0176 (5)	0.0211 (6)	0.0188 (6)	−0.0008 (5)	0.0094 (5)	−0.0001 (5)
O2	0.0258 (5)	0.0194 (4)	0.0259 (5)	0.0052 (4)	0.0112 (4)	0.0038 (4)
C2	0.0147 (5)	0.0190 (6)	0.0166 (6)	−0.0013 (4)	0.0065 (4)	−0.0031 (4)
O3	0.0285 (5)	0.0340 (5)	0.0187 (4)	0.0051 (4)	0.0111 (4)	−0.0002 (4)
C3	0.0147 (5)	0.0179 (6)	0.0209 (6)	−0.0038 (4)	0.0049 (5)	−0.0015 (5)
O4	0.0193 (4)	0.0218 (4)	0.0269 (5)	−0.0009 (3)	0.0140 (4)	0.0020 (3)
C4	0.0282 (7)	0.0187 (6)	0.0326 (7)	0.0055 (5)	0.0106 (5)	0.0020 (5)
O5	0.0236 (4)	0.0204 (4)	0.0297 (5)	−0.0009 (4)	0.0145 (4)	0.0033 (4)
C5	0.0195 (6)	0.0160 (6)	0.0228 (6)	0.0032 (4)	0.0113 (5)	0.0002 (5)
C6	0.0331 (7)	0.0262 (7)	0.0417 (8)	0.0001 (6)	0.0284 (6)	0.0036 (6)
C7	0.0207 (6)	0.0190 (6)	0.0147 (5)	0.0009 (5)	0.0077 (4)	0.0009 (4)
C8	0.0245 (6)	0.0220 (6)	0.0308 (7)	0.0055 (5)	0.0130 (5)	0.0076 (5)
C11	0.0161 (6)	0.0156 (5)	0.0207 (6)	0.0033 (4)	0.0097 (5)	0.0032 (5)
C12	0.0185 (6)	0.0200 (6)	0.0204 (6)	−0.0011 (5)	0.0079 (5)	−0.0011 (5)
C13	0.0234 (6)	0.0258 (6)	0.0222 (6)	0.0001 (5)	0.0134 (5)	0.0024 (5)
C14	0.0194 (6)	0.0241 (6)	0.0336 (7)	−0.0012 (5)	0.0135 (5)	0.0070 (5)
C15	0.0187 (6)	0.0211 (6)	0.0292 (7)	−0.0029 (5)	0.0059 (5)	−0.0017 (5)
C16	0.0204 (6)	0.0212 (6)	0.0207 (6)	0.0021 (5)	0.0078 (5)	−0.0006 (5)

### Geometric parameters (Å, °)

**Table:**

O1—C3	1.2106 (14)	C6—H6B	0.9600
N1—C7	1.3867 (15)	C6—H6C	0.9600
N1—C2	1.4031 (14)	C7—C8	1.5019 (16)
N1—C11	1.4466 (14)	C8—H8A	0.9600
C1—C2	1.3376 (16)	C8—H8B	0.9600
C1—C3	1.4672 (16)	C8—H8C	0.9600
C1—H1A	0.9300	C11—C16	1.3823 (16)
O2—C3	1.3413 (14)	C11—C12	1.3849 (16)
O2—C4	1.4418 (14)	C12—C13	1.3878 (15)
C2—C5	1.5088 (15)	C12—H12A	0.9300
O3—C5	1.2030 (14)	C13—C14	1.3820 (18)
O4—C5	1.3290 (14)	C13—H13A	0.9300
O4—C6	1.4432 (13)	C14—C15	1.3841 (18)
C4—H4A	0.9600	C14—H14A	0.9300
C4—H4B	0.9600	C15—C16	1.3876 (16)
C4—H4C	0.9600	C15—H15A	0.9300
O5—C7	1.2183 (14)	C16—H16A	0.9300
C6—H6A	0.9600		
			
C7—N1—C2	120.51 (9)	H6B—C6—H6C	109.5
C7—N1—C11	121.36 (9)	O5—C7—N1	120.25 (10)
C2—N1—C11	118.12 (9)	O5—C7—C8	122.84 (11)
C2—C1—C3	123.55 (10)	N1—C7—C8	116.91 (10)
C2—C1—H1A	118.2	C7—C8—H8A	109.5
C3—C1—H1A	118.2	C7—C8—H8B	109.5
C3—O2—C4	115.32 (9)	H8A—C8—H8B	109.5
C1—C2—N1	121.65 (10)	C7—C8—H8C	109.5
C1—C2—C5	122.21 (10)	H8A—C8—H8C	109.5
N1—C2—C5	115.69 (9)	H8B—C8—H8C	109.5
O1—C3—O2	123.64 (10)	C16—C11—C12	121.21 (10)
O1—C3—C1	126.44 (11)	C16—C11—N1	118.84 (10)
O2—C3—C1	109.87 (10)	C12—C11—N1	119.95 (10)
C5—O4—C6	114.63 (10)	C11—C12—C13	119.20 (11)
O2—C4—H4A	109.5	C11—C12—H12A	120.4
O2—C4—H4B	109.5	C13—C12—H12A	120.4
H4A—C4—H4B	109.5	C14—C13—C12	120.11 (11)
O2—C4—H4C	109.5	C14—C13—H13A	119.9
H4A—C4—H4C	109.5	C12—C13—H13A	119.9
H4B—C4—H4C	109.5	C13—C14—C15	120.12 (11)
O3—C5—O4	125.75 (11)	C13—C14—H14A	119.9
O3—C5—C2	121.56 (11)	C15—C14—H14A	119.9
O4—C5—C2	112.68 (10)	C14—C15—C16	120.35 (11)
O4—C6—H6A	109.5	C14—C15—H15A	119.8
O4—C6—H6B	109.5	C16—C15—H15A	119.8
H6A—C6—H6B	109.5	C11—C16—C15	119.00 (11)
O4—C6—H6C	109.5	C11—C16—H16A	120.5
H6A—C6—H6C	109.5	C15—C16—H16A	120.5

### Hydrogen-bond geometry (Å, °)

**Table:**

*D*—H···*A*	*D*—H	H···*A*	*D*···*A*	*D*—H···*A*
C4—H4C···O3^i^	0.96	2.53	3.0831 (16)	117.
C14—H14A···O3^ii^	0.93	2.57	3.2016 (15)	125.
C12—H12A···O5^iii^	0.93	2.51	3.3073 (15)	145.

Symmetry codes: (i) *x*−1/2, −*y*+1/2, *z*−1/2; (ii) *x*+1/2, −*y*+1/2, *z*−1/2; (iii) −*x*+1, −*y*, −*z*.
